# Bioactive Compounds of Ginger (*Zingiber officinale Roscoe*): Antimicrobial Potential and Microalgae-Based Encapsulation Strategies for Combating Biofilms

**DOI:** 10.3390/antibiotics15070642

**Published:** 2026-06-27

**Authors:** Malika Mekhalfi, Sabine Berteina-Raboin

**Affiliations:** Institut de Chimie Organique et Analytique (ICOA), Université d’Orléans, UMR-CNRS 7311, BP 6759, Rue de Chartres, CEDEX 2, 45067 Orléans, France

**Keywords:** *Zinziber officinale Roscoe*, synergetic effects, antimicrobial peptides, biofilm, microalgae

## Abstract

This review examines the bioactive compounds of ginger (*Zingiber officinale Roscoe*), with a particular focus on metabolites and antimicrobial peptides exhibiting antimicrobial activity. The chemical composition, biological properties, and mechanisms of action of the major ginger-derived compounds are discussed, with emphasis on their antibacterial, antifungal, anti-inflammatory, and antibiofilm potential. Particular attention is given to their antimicrobial spectrum and to potential synergistic interactions with other natural bioactive compounds that may enhance their efficacy against pathogenic microorganisms. Despite their promising therapeutic properties, the application of ginger-derived molecules against skin-associated pathogens remains challenging due to their limited stability, poor bioavailability, and the protective effects of microbial biofilms, which reduce treatment effectiveness and contribute to persistent infections. Current strategies designed to overcome these limitations, including chemical modification, liposomes, nanoemulsions, and hydrogel-based delivery systems, are reviewed. In addition, this review highlights the potential of microalgae-based encapsulation systems as innovative and sustainable platforms for the delivery of ginger bioactives. Owing to their diverse biochemical composition and structural characteristics, microalgae represent a promising source of natural biomaterials for the development of diverse encapsulation strategies. These emerging systems may potentially improve the stability, controlled release, bioavailability, and antibiofilm efficacy of ginger-derived compounds, supporting the development of novel formulations for the management of biofilm-associated skin infections.

## 1. Introduction

Ginger’s reputation and health benefits are well established in traditional Chinese medicine and Ayurveda. Mixtures of medicinal plants often yield greater therapeutic effects than single-plant preparations, a phenomenon known as synergy; however, such combinations can also be harmful, which underscores the need for rigorous evaluation of their efficacy and safety. The part of ginger (*Zingiber officinale Roscoe*, family *Zingiberaceae*) recognized as medicinal is the rhizome. *Zingiber* is recommended for numerous conditions and, as such, could be considered a broad-spectrum remedy. It can be used alone, fresh or dried, and more broadly combined with other plants [1]. Its initial applications concerned digestive disorders [2,3]; subsequently, various other effects were identified [4]. The volatile oils contained in the rhizomes account for approximately 2 to 3% of their composition and are often marketed as alcoholic extracts for their analgesic, anti-inflammatory, hepatoprotective, and nephroprotective properties, as well as their antioxidant effects, to name just a few, though other applications are also claimed [5,6,7,8,9]. The chemical composition of ginger rhizomes, which varies according to their geographical origin and storage conditions, has been extensively reviewed by Mahboubi et al. [9]. In addition to the promising compounds it contains, there are numerous examples of the synergistic effects of other compounds. Indeed, the increasing use of combination therapy is being applied across various therapeutic areas because many diseases involve multiple pathways of action that need to be targeted simultaneously. Wagner et al. [10] discussed the demonstration of the synergistic efficacy of a given mixture and noted that Berenbaum’s “isobole method” [11] is one of the simplest and most effective. Among these synergistic combinations, gallocatechins from green tea and curcuminoids from turmeric have been reported to enhance the biological activity of ginger-derived compounds. Among the interesting compounds found in these plants are polyphenols, which have demonstrated potential against various therapeutic targets, including anti-inflammatory and anticancer applications. To cite one well-known example, epigallocatechin gallate (*Camellia sinensis*) helps maintain the activity of penicillin against *Staphylococcus aureus* [12,13]. In fact, this blocks the efflux pump system found in many bacteria, thereby preventing either the entry of agents into the bacterium or the expulsion of antibiotics that have already penetrated the bacterial cell. Furthermore, plants generally have fewer side effects [14,15]. In addition to the synergistic effect that a combination of plants can have on the various causes of a given condition, one can also observe an increase in activity through improved bioavailability or biodistribution of the active compounds. Beyond synergistic combinations with other medicinal plants, improving the stability and bioavailability of ginger-derived compounds remains a major challenge. In this regard, natural encapsulation systems, including those based on microalgae-derived biomaterials, are emerging as promising strategies to enhance the therapeutic potential of ginger bioactives. The present review provides an integrative overview of ginger bioactive compounds by synthesizing recent advances in their antimicrobial and antibiofilm activities, synergistic interactions, and antimicrobial peptides. Particular emphasis is placed on the therapeutic limitations of ginger-derived compounds, including poor stability, limited bioavailability, and biofilm-associated resistance, as well as on emerging microalgae-based encapsulation and drug-delivery strategies designed to overcome these challenges. By integrating these aspects, this review highlights the potential of microalgae-derived biomaterials as promising platforms for enhancing the stability, bioavailability, and antibiofilm efficacy of ginger-derived compounds.

## 2. Discussion

### 2.1. Phytochemical Composition and Biological Activities of Z. officinale Roscoe

**Figure 1:**
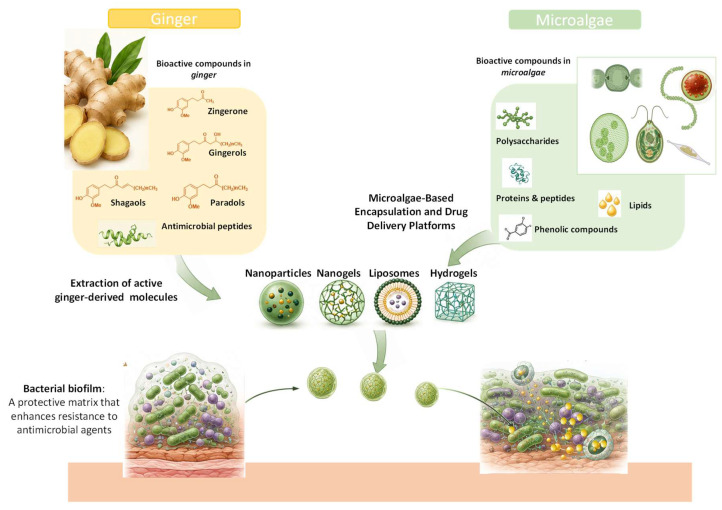
Ginger bioactive compounds: from antimicrobial activity to microalgae-based encapsulation.

The composition of ginger has been studied for several decades; it depends on cultivation methods and locations, whether the ginger is fresh or dried, and the extraction processes used. Geographical origin, together with cultivation practices and post-harvest processing, is one of the major factors influencing the chemical composition of ginger. A comprehensive overview of the phytochemical composition of *Z. officinale Roscoe* and the biological activities of its major constituents was provided by Li et al. in 2019 [16]. Ginger [17,18,19] belongs to the same botanical family as turmeric (*Curcuma longa* L.) and cardamom (*Elettaria cardamomum* (L.) *Maton.*) [20,21]. It has been reported to act a synergistically with turmeric and other botanicals, enhancing their biological activities (Figure 1).

Turmeric contains curcuminoids, phenolic compounds that are partly analogous to the gingerols and shogaols found in ginger [22] and share similar properties. Cardamom (*Elettaria cardamomum*) contains 1,8-cineole and catechins, the latter also being present in tea. 1,8-Cineole (eucalyptol), which is also found in ginger, is a multifunctional compound known for its aromatic, antimicrobial and anti-inflammatory properties [23,24].

Among the compounds most commonly associated with the therapeutic activity of *Z. officinale Roscoe.* are gingerols, a family of phenolic ketones that occur in relatively high concentrations and are largely responsible for the characteristic pungent flavour of ginger [16,25]. They are generally converted into their respective shogaols through dehydration, particularly during long-term storage, drying, or heat treatment. These structural modifications are responsible for the differences observed in their biological activities [26,27]. The corresponding paradols, meanwhile, are obtained by the hydrogenation of shogaols [16,25]. Other compounds are also present in smaller amounts, including zingerone, a degradation product of the heat-labile 6-gingerol (*n* = 6), as well as vitamins (notably C and B6) and minerals.

**Figure 2:**
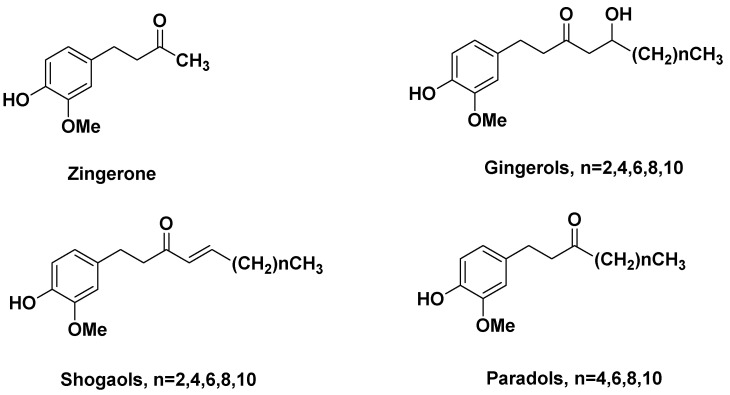
Compounds found in *Z. officinale Roscoe*.

Gingerols exhibit a broad range of biological activities, including antioxidant, anti-inflammatory, antimicrobial, and antitumor effects. Similarly, zingerone possesses antioxidant, anti-inflammatory, and antimicrobial activities. Shogaols, which are formed through the dehydration of gingerols, are also recognised for their antioxidant and antitumor activities, whereas the corresponding hydrogenated paradol-type derivatives exhibit antioxidant, antitumor, and antibacterial activities (Figure 2).

The primary benefit of ginger, as recognized by traditional Ayurvedic and Chinese medicine, is its ability to alleviate nausea. In addition to its antiemetic effects, several ginger constituents exhibit hepatoprotective and gastroprotective activities, which may contribute to the alleviation of gastrointestinal discomfort [28,29].

The rapid emergence of antimicrobial resistance among bacterial pathogens has become a major global health challenge, limiting the effectiveness of many currently available antibiotics and other antimicrobial therapies [30]. Therefore, ginger and its bioactive compounds have attracted considerable interest as potential alternatives or adjuncts to conventional antimicrobial therapies. Numerous studies have demonstrated the antimicrobial activity of ginger against a wide range of pathogens, including *Escherichia coli*, *Salmonella typhi*, *Candida albicans* and *Mycobacterium tuberculosis*. Beyond its antimicrobial properties, ginger also exhibits potent anti-inflammatory activity. Several in vivo studies have shown that ginger and its bioactive constituents reduce the expression of pro-inflammatory cytokines and modulate inflammatory signaling pathways [31]. The active compounds in ginger, such as gingerols, shogaols, and certain diterpenoids, have been reported to have anti-serotonergic effects and act as 5-HT_3_ receptor antagonists [32].

Some gingerols have also been shown to inhibit inflammatory processes and hyperproliferation, which are involved in carcinogenesis, angiogenesis, and metastasis formation [33,34]. Ginger and its bioactive compounds have shown promising anticancer effects in several gastrointestinal cancers, including pancreatic cancer, although further clinical studies are needed to confirm their therapeutic potential [35].

Clinical evidence also supports the anti-inflammatory effects of ginger and its bioactive constituents. With regard to its anti-inflammatory activity, it is worth noting the potential benefits of ginger extract supplementation in patients with knee osteoarthritis. It has been shown to have analgesic and functional effects in reducing pain and improving mobility. These initial clinical studies were conducted against a placebo and should be continued to confirm the benefits of this supplementation [36,37,38].

**Figure 3:**
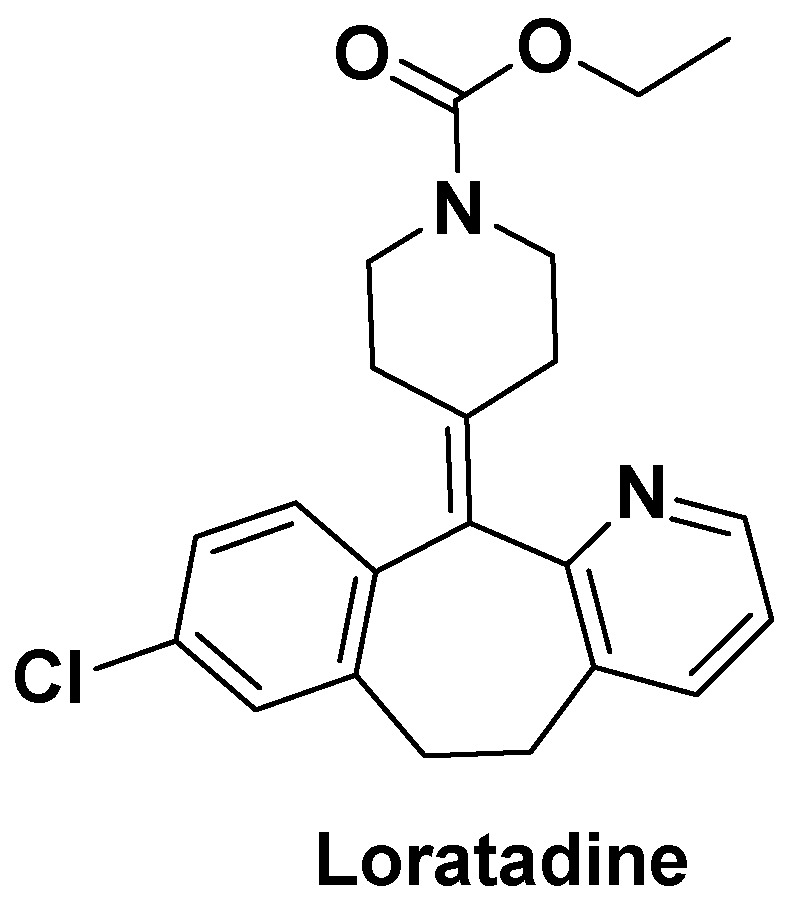
Loratadine structure.

The antioxidant properties of *Z. officinale Roscoe* help reduce the formation of reactive oxygen species (ROS) and thus alleviate oxidative stress, thereby supporting the body’s natural antioxidant defenses. It is now well established that the process of oxidative stress can lead, in the long term, to numerous chronic diseases, such as rheumatoid arthritis, as well as chronic inflammation and cancer. These antioxidant properties may contribute to the prevention or mitigation of several chronic disorders associated with oxidative stress and inflammation, including cardiovascular diseases and rheumatoid arthritis, although further clinical evidence is required. In a clinical study, an extract of *Z. officinale Roscoe* showed efficacy comparable to that of loratadine (Figure 3), a histamine H1 receptor antagonist, in patients with allergic rhinitis [39,40].

### 2.2. Synergistic Antimicrobial Effects of Z. officinale Roscoe and Related Species

In 2017, Bereksi et al. [41] studied several plants, including *Z. officinale Roscoe*, either alone or in combination with various antibiotics. The methanolic extracts were evaluated against a panel of Gram-positive and Gram-negative pathogenic bacteria, including *S. aureus*, *Enterococcus faecalis*, *E. coli*, *Klebsiella pneumoniae*, and *Pseudomonas aeruginosa*. The authors demonstrated a synergistic interaction between cefazolin and *Z. officinale Roscoe* extract, resulting in enhanced antibacterial activity. However, the extracts alone exhibited relatively weak antibacterial activity, which may be related to limited penetration or insufficient concentrations of the active compounds. Similar synergistic interactions between antibiotics and plant extracts have been reported previously [42], with several studies showing that such combinations may reduce bacterial resistance and enhance antimicrobial efficacy [43,44].

These various studies highlight different types of interactions that may occur either between plant extracts and conventional antibiotics or among combinations of medicinal plants. Depending on the compounds involved and their concentrations, these interactions may be synergistic, additive, antagonistic, or neutral, the latter indicating no significant improvement compared with the antibiotic or plant extract used alone. Synergistic interactions correspond to combinations producing a greater antimicrobial effect than expected from the individual compounds, whereas additive interactions produce effects close to the sum of the individual activities. Indifferent (or neutral) interactions do not significantly alter antimicrobial activity, while antagonistic interactions result in reduced efficacy compared with the compounds used separately [45]. In most in vitro studies, the evaluation of these interactions relies primarily on the measurement of inhibition zones and changes in minimum inhibitory concentration (MIC) values. A synergistic interaction is generally characterized by an increase in antimicrobial efficacy, reflected by a reduction in the MIC values of the compounds used in combination compared with their individual administration.

**Figure 4:**
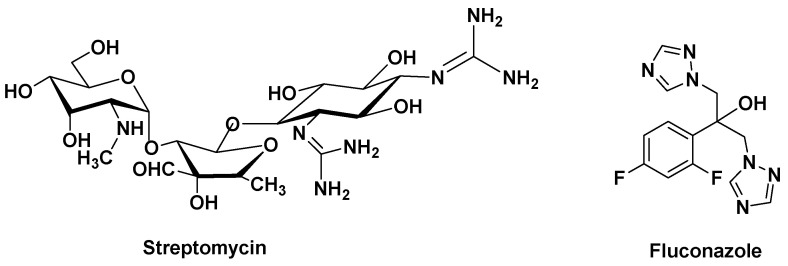
Streptomycin and Fluconazole structures.

In 2023, Gunathilake et al. [46] evaluated the antimicrobial activity of essential oils and oleoresins derived from three spices, including *Z. officinale Roscoe* against five bacteria (*E. coli*, *P. aeruginosa*, *S. aureus*, *Bacillus subtilis*, *Salmonella typhimurium*) and two fungi (*Aspergillus niger*, *C. albicans*) then in combination with commercially available antimicrobials. Antimicrobial activity was assessed here by agar disk diffusion and by determining the MIC. These results were compared to those of two commercial antibiotics, streptomycin and fluconazole (Figure 4). The results showed that essential oils exhibited stronger antimicrobial activity than oleoresins and that remarkable synergies were observed for the mixture of cinnamon oil and ginger essential oil against the Gram-positive bacterium *Bacillus subtilis*. The same is observed for the mixture of clove buds and ginger against *Aspergillus niger* (a filamentous fungus). Conversely, other combinations showed antagonistic, indifferent, or even additive effects. These findings illustrate that combining plant-derived products can enhance antimicrobial efficacy, although the outcome strongly depends on the specific combination and target microorganism. An important advantage of such synergistic interactions is the possibility of achieving antimicrobial efficacy at lower effective doses, which may reduce toxicity and limit the development of resistance.

Besides *Z. officinale Roscoe*, other species of the genus *Zingiber* have also shown promising synergistic antimicrobial properties. In a 2017 study, Narachai et al. [47] investigated for the first time the antibacterial and synergistic activities of the essential oil of *Z. cassumunar* against extensively drug-resistant (XDR) strains of *Acinetobacter baumannii*, an important nosocomial pathogen. Using disk diffusion and resazurin assays, the essential oil exhibited significant antibacterial activity, with MIC and MBC values less than 10 mg/mL and complete bacterial inhibition occurring within one hour. Combinations with several antibiotics (gentamicin, amikacin, ciprofloxacin, levofloxacin, a β-lactams and tetracyclines) revealed clear synergistic effects despite the strains’ resistance profiles. These findings highlight the potential of *Z. cassumunar* as a natural adjuvant for combating antibiotic-resistant infections, particularly those caused by multidrug-resistant nosocomial pathogens. Similarly, Singh et al. [48] evaluated the synergistic effects of clove, eucalyptus, and ginger extracts combined with conventional antibiotics against multidrug-resistant *P. aeruginosa*. Among the antibiotics tested, ciprofloxacin exhibited the strongest activity against *P. aeruginosa*, followed by ceftazidime and gentamicin, whereas clove showed the highest antibacterial activity among the plant extracts. Notably, a significant synergistic interaction was observed between ceftazidime and the clove–ginger combination, resulting in more than a two-fold reduction in the MIC.

In 2022, Saxena et al. [49] investigated the antifungal activity of essential oils from *Z. officinale Roscoe* and *Curcuma longa* L. against dermatophytes responsible for zoonotic dermatomycosis. The chemical composition of the oils was characterised by GC-MS prior to biological evaluation. Their combined dermatophytic activity was evaluated against *Trichophyton verrucosum* and *Microsporum canis*. The combination exhibited markedly stronger antidermatophytic activity than either oil used alone and outperformed conventional antifungal agents such as clotrimazole and ketoconazole. These results further support the synergistic potential of ginger and turmeric and suggest that their combination could represent an effective natural alternative for the treatment of zoonotic dermatophytosis. Although essential oils generally require relatively high concentrations to achieve fungistatic effects [50], the synergistic interaction between *C. longa* L. and *Z. officinale Roscoe* may allow the use of lower effective doses, a benefit that has also been reported when these oils are combined with conventional antifungal drugs [51].

Numerous other studies have reported the biological activities of ginger, either alone or in combination with other natural products or conventional drugs. These synergistic interactions may enhance therapeutic efficacy and reduce the doses required to achieve antimicrobial activity. Although these effects have been extensively reviewed elsewhere. In contrast, comparatively less attention has been paid to the antimicrobial peptides (AMPs) naturally present in ginger. This emerging field represents a promising avenue for the development of novel strategies to combat antimicrobial resistance [52].

### 2.3. Ginger-Derived Antimicrobial Peptides (AMPs)

Antimicrobial peptides, small fragments of amino acids typically comprising between 3 and 50 residues, have attracted considerable interest in recent years due to their broad-spectrum efficacy against multidrug-resistant pathogens [52]. Their amino acid composition, three-dimensional structure and biological origin are major determinants of their functional properties, making them promising alternatives to conventional antibiotics. Among plant-derived AMPs, those isolated from ginger (*Z. officinale Roscoe*) have recently attracted increasing attention owing to their diverse biological activities and potential therapeutic applications [53]. A comparative peptidomic analysis of *Z. officinale Roscoe* showed that fractions smaller than 3 kDa exhibited the highest antioxidant activity and enabled the identification of several peptide sequences by HPLC-MALDI-ToF MS [53].

These peptides are associated with metabolic and hydrolytic functions, as well as antimicrobial and antifungal activities, reinforcing the value of ginger as a source of bioactive peptides. Similarly, Sunna et al. [54] subjected ginger proteins to enzymatic hydrolysis, identified the resulting peptides by LC-MS/MS, screened the resulting peptide pool in silico, and selected 41 peptides for molecular docking, SPPS synthesis and in vitro testing. Several peptides exhibited bacteriostatic activity and inhibited angiotensin-converting enzyme (ACE). Notably, isolated peptides often outperformed crude hydrolysates, suggesting that the identification and selective production of individual AMPs may facilitate their functional characterisation and future therapeutic development. Further investigation of these relatively understudied plant-derived peptides could facilitate the discovery of new bioactive compounds from other rhizomatous species with reported medicinal properties.

### 2.4. Antimicrobial Activity of Ginger-Derived Compounds

Ginger contains numerous bioactive compounds with a wide range of biological activities, including antibacterial effects [46]. The antimicrobial activity of ginger-derived compounds is mediated through several complementary mechanisms that target microbial growth, virulence, and biofilm formation. For instance, ginger essential oil has been shown to increase bacterial cell membrane permeability, thereby enhancing the susceptibility of pathogens such as *Salmonella typhi*, *S. epidermidis*, and *Streptococcus mutans* [55]. Moreover, major phenolic compounds such as 6-gingerol and 6-shogaol exhibit antibacterial activity against clinically relevant pathogens including *P. aeruginosa*, *A. baumannii*, and *Klebsiella pneumoniae* (MIC ≈ 0.4 mg/mL), as well as *S. aureus* (MIC ≈ 0.2 mg/mL) [56]. These compounds have also been reported to inhibit genes involved in biofilm formation, such as *ECE1* and *HWP1*, in the fungal pathogen *C. albicans* [57]. In addition, they interfere with cell-to-cell communication (quorum sensing), thereby reducing microbial virulence [58,59].

Shogaol derivatives have demonstrated significant antimicrobial activity against foodborne pathogens, with reported MIC values as low as 62.5 µg/mL for *Klebsiella pneumoniae* (ATCC 9633) [60]. Furthermore, these compounds may inhibit key metabolic enzymes by disrupting growth-related enzymatic pathways, in both bacteria and fungi, for example, through the inhibition of enzymes such as α-amylase [56,61]. These effects are likely related to the reactive nature of ginger-derived phenolic compounds, which can interact with cellular proteins and membranes. Overall, these findings highlight the multifactorial nature of the antimicrobial activity of ginger-derived compounds. However, most of the available data are derived from in vitro studies, and further in vivo investigations are required to confirm their therapeutic potential [62].

### 2.5. Limitations and Challenges in the Therapeutic Application of Antimicrobial Ginger-Derived Compounds

Despite their promising antimicrobial effects, the therapeutic application of ginger-derived compounds against resistant pathogens remains challenging. Their clinical and industrial applications are constrained by physicochemical and pharmacokinetic limitations. These compounds often exhibit limited chemical stability and low aqueous solubility, which can reduce both systemic and local bioavailability [63,64]. These limitations are further exacerbated in the context of bacterial biofilm formation, where the extracellular polymeric matrix acts as a barrier to the diffusion and penetration of antimicrobial agents. This contributes to increased microbial tolerance and may promote the development of antibiotic resistance [65]. For example, biofilms formed by skin-associated pathogens such as *Cutibacterium acnes,* which play an important role in acne pathogenesis, exhibit complex structural and biochemical organization. These matrices are composed of polysaccharides, proteins, and extracellular DNA, which further limit the penetration and effective concentration of antimicrobial agents within the biofilm [66].

To overcome these limitations, strategies aimed at improving the stability, solubility, and bioavailability of ginger-derived compounds have been actively investigated. Several approaches have been explored to enhance the physicochemical properties and therapeutic potential of ginger-derived compounds, including structural modification and advanced delivery systems. However, because the present review focuses on natural compounds and sustainable formulations, particular attention is given to encapsulation-based strategies.

Among the various approaches proposed to enhance the therapeutic efficacy of ginger-derived compounds, encapsulation-based delivery systems have emerged as one of the most promising strategies. Curcumin provides a representative example, as its clinical application is limited by poor aqueous solubility and chemical instability. Strategies such as co-administration with piperine or encapsulation in phospholipid-based carriers have been developed to overcome these limitations. Encapsulation has been shown to protect active compounds from degradation, improve their solubility in biological media, and modulate their release kinetics at the site of action, thereby increasing the likelihood of achieving effective local antimicrobial concentrations, including within biofilms [67].

Various encapsulation strategies have been widely investigated to optimize the delivery of ginger-derived compounds. Liposomes constitute a promising approach, as they can encapsulate both hydrophobic and amphiphilic molecules, enhance skin penetration, and allow functionalization for targeted delivery [68]. Nanoemulsions improve the solubility and dispersion of hydrophobic compounds while providing colloidal stability suitable for topical formulations. Polymeric nanoparticles enable precise control of release kinetics through mechanisms involving diffusion and degradation, and can be functionalized with ligands or anti-biofilm agents [69]. Hydrogels also constitute a relevant strategy, as they form adhesive three-dimensional matrices capable of promoting sustained release and creating a microenvironment conducive to healing, making them particularly suitable for the local treatment of skin biofilms [70].

For ginger-derived compounds, encapsulation strategies have demonstrated significant potential in overcoming their intrinsic physicochemical limitations [71]. Several studies have investigated the encapsulation of ginger-derived bioactive compounds in natural biopolymers, such as polysaccharides (e.g., gum arabic) [72] and protein-based matrices [73]. These approaches have been shown to improve physicochemical properties, including stability and solubility, while maintaining or even enhancing biological activity [71]. For instance, the microencapsulation of red ginger essential oil using gum arabic as a wall material has retained significant antibacterial activity against *E. coli* and *S. aureus*. In this context, reductions in bacterial counts of approximately 1.8 and 2.3 log CFU g^−1^ were reported for *E. coli* and *S. aureus*, respectively, further supporting the ability of encapsulation to preserve and potentially enhance antimicrobial efficacy [72].

Overall, the selection of an appropriate carrier matrix is a key factor in determining the stability, bioavailability, and antimicrobial efficacy of encapsulated bioactive compounds. Consequently, increasing attention has been directed toward natural biomaterials that combine biocompatibility, sustainability, and multifunctional properties.

## 3. Microalgae as a Promising Source of Encapsulation and Drug Delivery Materials

Among emerging delivery strategies, microalgae-derived biomaterials have attracted increasing attention due not only to their biocompatibility and structural versatility [74] but also to their biodegradability, sustainability, and rich biochemical composition, including polysaccharides, proteins, and lipids [75,76]. These unique characteristics distinguish microalgae from many conventional biomaterial sources and make them particularly attractive for the development of next-generation delivery systems [77]. Importantly, microalgal biomaterials generally exhibit low toxicity, excellent biocompatibility, and tunable release properties, all of which are highly desirable features for drug delivery applications [77]. Moreover, some microalgal biomolecules exhibit intrinsic bioactivities such as antioxidant, anti-inflammatory, and mucoadhesive properties, which may further enhance the stability, retention, and bioavailability of encapsulated compounds [78,79]. Consequently, microalgae-derived biomaterials represent promising carriers for the encapsulation of bioactive compounds with limited stability and solubility, including ginger-derived molecules [77,78,79,80].

Microalgae-derived biomaterials offer several concrete strategies for drug delivery system design. For example, extracellular polysaccharides (EPS) and sulfated polysaccharides produced by microalgae are widely employed to fabricate hydrogels, microspheres, and nanoparticulate systems that encapsulate bioactive compounds and enable controlled release [81]. In addition, whole microalgal cells can act as natural carriers, protecting encapsulated compounds from chemical and enzymatic degradation during delivery [74]. Furthermore, the large specific surface area of microalgae enables the effective adsorption and binding of bioactive molecules, thereby enhancing delivery efficiency [82]. More advanced approaches use hybrid systems combining microalgae with nanomaterials, which have been shown to improve drug loading and therapeutic performance [75].

These properties highlight the feasibility and potential of microalgae-based systems as multifunctional delivery platforms, capable of improving the stability, bioavailability, and antimicrobial efficacy of compounds such as those derived from ginger [75].

Although direct applications of microalgae-derived biomaterials for encapsulating ginger extracts and ginger-derived compounds remain limited, their unique composition and multifunctional properties make them attractive candidates for advanced drug delivery applications [77]. Consequently, microalgae offer a renewable, biocompatible alternative to conventional plant or animal-derived materials, with added advantages such as biodegradability, structural versatility, and intrinsic bioactivities [83].

Notably, a recent study described a nanocomposite combining *Chlorella vulgaris* and 6-gingerol that exhibited promising anticancer and antimicrobial activities, highlighting the potential of microalgae-based platforms for ginger-derived molecules. However, dedicated studies are still needed to establish microalgae-derived encapsulation systems specifically tailored for ginger extracts and ginger-derived compounds and to evaluate their loading efficiency, physicochemical stability, and controlled-release performance [80].

In parallel, extracellular polysaccharides (EPS), particularly abundant in red microalgae such as *Porphyridium purpureum*, have been extensively studied for their ability to form gel-like and mucoadhesive matrices that enable controlled release of encapsulated compounds [84,85]. These EPS exhibit highly hydrated and viscoelastic structures, enabling them to act as natural carriers for bioactive molecules and to modulate their release profiles. Similarly, protein-rich microalgae such as *Arthrospira platensis* provide proteins and bioactive peptides with interesting functional and biological properties, making them promising candidates for the development of colloidal and nanoparticle-based delivery systems [86].

In addition, diatom microalgae such as *Thalassiosira pseudonana* provide a distinct strategy based on their silica-based frustule architecture. These naturally occurring nanoporous structures can act as carriers for bioactive compounds, offering high loading capacity and controlled release profiles. Their rigid structure also protects encapsulated molecules and allows surface functionalization, making them promising candidates for advanced drug delivery applications [87]. Beyond their structural role in drug delivery systems, microalgae also produce bioactive compounds such as C-phycocyanin, carotenoids, and chlorophyll derivatives, which exhibit antioxidant and anti-inflammatory properties. These molecules may complement the activity of ginger-derived compounds and contribute to enhanced therapeutic efficacy. In practice, microalgae-derived biomaterials may serve as encapsulation matrices, either as purified biopolymers or enriched extracts, to improve the physicochemical compatibility, stability, and release profiles of ginger-derived compounds [76]. These systems aim to combine the protective and delivery functions of the encapsulation matrix with the antimicrobial activity of ginger-derived bioactive compounds, while simultaneously exploiting the intrinsic biological properties of the microalgal carrier. Despite these promising advances, several challenges remain before microalgae-derived biomaterials can be fully exploited as delivery systems for ginger-derived compounds. Future research should therefore focus on validating and optimizing these systems through integrated experimental and computational approaches. This includes physicochemical characterization of the formulations, assessment of antimicrobial activity using parameters such as MICs and minimum biofilm eradication concentrations (MBECs), as well as penetration studies employing confocal microscopy or ex vivo skin models. Appropriately designed in vivo studies will also be required to evaluate safety, efficacy, and pharmacokinetic behavior. Furthermore, in silico modeling of diffusion and release kinetics could provide valuable insights for predicting and optimizing the antibacterial performance of encapsulated ginger-derived compounds.

## 4. Conclusions

These compounds exhibit considerable potential as antimicrobial and antibiofilm agents for topical applications, complementing conventional treatments. Their anti-inflammatory, antioxidant and antimicrobial effects, including those mediated by recently described antimicrobial peptides (AMPs), warrant in-depth evaluation of their preventive and synergistic benefits. However, their clinical translation remains limited by reduced stability and low bioavailability following oral or topical administration. Furthermore, biofilms protect pathogens and reduce the efficacy of the compounds. Despite the growing number of studies reporting the antimicrobial properties of ginger-derived compounds, several limitations remain. Most investigations are based on in vitro models, whereas standardized in vivo studies and clinical trials remain scarce. In addition, variations in extraction methods, phytochemical composition, and experimental protocols make direct comparisons between studies difficult. It is therefore necessary to conduct standardised preclinical and clinical studies to assess the efficacy, safety and drug interactions of standardised extracts. At the same time, research into encapsulation and delivery strategies must be stepped up to improve stability, controlled release and anti-biofilm penetration. In this context, microalgae-based encapsulation systems represent a promising avenue. Thanks to their rich biochemical composition, biocompatibility, and favorable physicochemical properties, these natural matrices could enable the co-encapsulation of several ginger-derived bioactive compounds, improve their stability and optimise their release, whilst potentially enhancing their activity within biofilms. Finally, epidemiological studies and randomised trials on standardised supplementation could clarify the preventive impact of regular ginger consumption on microbial infections, oxidative stress and chronic inflammation.

## Data Availability

No new data were created or analyzed in this study.
